# Push-Out Bond Strength of Glass Fiber Endodontic Posts with Different Diameters

**DOI:** 10.3390/ma17071492

**Published:** 2024-03-25

**Authors:** Zsolt Rajnics, Dávid Pammer, Anikó Kőnig-Péter, Kinga Turzó, Gyula Marada, Márta Radnai

**Affiliations:** 1 Faculty of Medicine, Dental School, University of Pécs, 7623 Pécs, Hungary; turzo.kinga@pte.hu (K.T.); marada.gyula@pte.hu (G.M.); 2 Department of Materials Science and Engineering, Faculty of Mechanical Engineering, Budapest University of Technology and Economics, 1111 Budapest, Hungary; dav.pammer@gmail.com; 3 Institute of Bioanalysis, Medical School, University of Pécs, 7624 Pécs, Hungary; aniko.konig@aok.pte.hu; 4 Department of Prosthodontics, Faculty of Dentistry, University of Szeged, 6720 Szeged, Hungary; martaradnai@yahoo.com

**Keywords:** fiber posts, endodontic posts, prefabricated posts, push-out bond strength, PBS

## Abstract

(1) Background: The retention of intraradicular posts is an important factor for the prognosis of endodontically treated teeth. The purpose of this study was to evaluate the push-out bond strength (PBS) of the posts relating to their diameter and region of the root. (2) Methods: A total of 40 premolar teeth (decoronated and root canal-filled) were divided into four groups (n = 10). After post-space preparation, different sizes (1.0, 1.2, 1.5, and 2.0 mm) of glass fiber posts were luted with resin cement into the root canals. After placement, 2 mm thick slices were cut from the roots according to their apical, middle, and coronal regions (n = 116). Push-out tests were carried out in a universal testing machine on each slice. A statistical evaluation of the data was applied. (3) Results: When comparing the diameter, the 2.0 mm posts had the highest PBS (111.99 ± 10.40 N), while the 1.0 mm posts had the lowest PBS (99.98 ± 8.05 N). Divided by the surface of the bonded area, the average PBS value was the highest for the 1.0 mm posts (18.20 ± 1.67 MPa) and the lowest for the 2.0 mm posts (12.08 ± 1.05 MPa). (4) Conclusions: Within the limitations of the study, when comparing the regions of the roots, no significant differences were found among the PBS values of the three regions (*p* = 0.219). When comparing the diameters, significant differences were shown between the PBS values of the four groups (*p* = 0.023 and *p* = 0.003, respectively).

## 1. Introduction

Following endodontic treatment, the preservation and further restoration of root canal-filled teeth requires crucial decisions [1]. These teeth have a higher chance of fracture (with consequent tooth loss) compared to vital teeth, especially at a subgingival location [2]. Preparation and endodontic procedures further weaken the tooth via internal structure loss, leading to cusp deflection during function [3]. Intraradicular posts and core buildup may be used to reconstruct root canal-filled teeth when there is more than 50% coronal structure loss, and three or more axial walls of the clinical crown is absent due to caries, previous restorations, or fractures [4]. The aim of their usage is to provide both an apical and coronal closure of the root canal to protect and maintain the remaining intact tooth structure, providing a stable foundation and support for the definitive restoration (i.e., fixed restorations), also restoring their lost biological function (i.e., mastication, phonetics, and aesthetics) [4,5].

Traditionally, posts can be differentiated into two main categories: individually made and prefabricated posts [1]. These can be further categorized by several factors, such as fixation (active or passive), shape (tapered or parallel), material composition, etc. [1]. Individual posts require multiple appointments to be made; therefore, the temporary protection of the root canal is required after coronal and intraradicular preparation. The risk of root canal reinfection due to coronal leakage can occur during the restoration of the tooth [6]. The prefabricated posts (i.e., metal, zirconia, fiber, composite, etc.) can be luted immediately in one appointment [4,5,6]. The chance of reinfection can be dramatically reduced by using sufficient isolation (e.g., rubber dam) during post-luting [7]. Fiber-reinforced composite (FRC) posts luted with resin cements are widely chosen to restore root canal-filled teeth [1]. FRC posts can be classified by the types of fibers embedded in the epoxy or methacrylate matrix: carbon, quartz, silica, or glass [8].

Scientific reviews are not unified on the survival rates of different post types. Some studies show higher survival rates for fiber-reinforced posts compared with prefabricated or custom metal posts [9], with no difference in the success rates, debonding, or root fractures [9,10]. Others found no statistically significant differences between their survival rates, considering a reliable option to use when post placement is indicated [11]. The fatigue resistance of FRC posts differs by structural characteristics (fiber diameter, fiber density, and fiber coverage on the post surface) and is highly influenced by the manufacturing process [12]. Furthermore, the type of resin matrix used to embed the fibers influences the flexural strength (FS). Different fibers with the same matrix embedding have a similar FS [13]. The most common failure possibility of root canal-filled teeth restored with FRC posts is mainly caused by the debonding of the post at the post-cementation root canal wall interface [7,14]. Post retention within the root canal is affected by several factors: the type of post (material, diameter, and form), the adhesive and cementation system, the operative procedures (absolute isolation of the tooth), and the configuration of the root canal system [1,7,14,15]. Recently, silica microfiber reinforced posts with unique characteristics have been available on the market. They have a hollow central cylindrical canal, which is extended along the whole length of the post, acting as a carrier, allowing their insertion and filling with composite cement in one step. Their mechanical characteristics are similar to those of traditional fiber posts [16].

The intention of this study was to evaluate the retention of glass fiber-reinforced posts (Rebilda Post, VOCO GmbH, Cuxhaven, Germany) with different diameters in different regions of the root canal with push-out tests. The first null hypothesis stated that with different post diameters, the push-out bond strength (PBS) will be the same. The second null hypothesis stated that along the length of the root canal, the PBS is equally distributed.

## 2. Materials and Methods

### 2.1. Tooth Selection and Specimen Preparation

In this study, forty single straight-rooted, caries-free mandibular premolar teeth were used. The teeth were extracted for orthodontic purposes and collected at the Dentoalveolar Division, Department of Dentistry, Oral and Maxillofacial Surgery, University of Pécs, Hungary. The inclusion criteria included the absence of resorptions and fractures of the root. All the selected teeth were fully developed (closed apex) and had a minimum length of 14 mm with straight root canals. Therefore, the morphology of the root canals allowed for the placement of at least 10 mm or longer prefabricated glass fiber-reinforced composite posts. The extracted teeth were cleaned with water and disinfected with a 6% formaldehyde solution for one hour and stored in distilled water at 37 °C for 24 h.

The crowns of the specimens were separated from the roots at the cemento-enamel junction using a 0.5 mm thick diamond-coated slow-speed band saw (Hager and Meisinger GmbH, Neuss, Germany) under copious water-cooling. To standardize the root canal lengths, all the roots were cut to 14 mm in length. The pulp tissue was removed with a #15 size nickel–titanium Donaldson file (VDW GmbH, Munich, Germany). The root canals were prepared starting with an ISO-size 10 nickel–titanium K-file (VDW GmbH, Munich, Germany) at the working length, which was visually determined by subtracting 1 mm from the length of the instrument placed at the apical foramen. The root canals were instrumented with ISO-size #15, #20, #25, #30, #35, and #40 nickel–titanium K-files (VDW GmbH, Munich, Germany). After reaching the #40 size at the apical stop, a step-back preparation was performed for each root canal with ISO-size #45, #50, #55, and #60 nickel–titanium K-files (VDW GmbH, Munich, Germany). In between the steps of root canal enlargement, 2 mL of 5.25% sodium hypochlorite was used to remove the debris and to clean and disinfect the root canal. After preparation, the root canals were dried using ISO-size #40 paper points (Shanghai Dochem Industries, Shanghai, China) and filled with gutta-percha cones (Shanghai Dochem Industries, Shanghai, China) and AH Plus resin sealer (Dentsply York PA, USA) using a standard lateral condensation technique with finger spreaders (VDW GmbH, Munich, Germany). After the endodontic treatment, cervical root canal cavities were filled with light-curing temporary filling material (Clip F, VOCO GmbH, Cuxhaven, Germany), and the roots were stored for 3 days at 37 °C in 100% relative humidity.

### 2.2. Post-Space Preparation and Post Cementation

The roots were randomly divided into four groups, having 10 roots in each group according to the diameter of the post (Rebilda Post, VOCO GmbH, Cuxhaven, Germany): 1.0 mm (group 1), 1.2 mm (group 2), 1.5 mm (group 3), and 2.0 mm (group 4). The gutta-percha was removed with #3 Gates-Glidden burs (VDW GmbH, Munich, Germany), except for 4 mm in the apical third. Each root canal was prepared at a working length of 10 mm from the sectioned surface. Table 1 shows the instrumentation used in each specific group, following the instructions of the manufacturer. During instrumentation, the root canals were rinsed with 2 mL of distilled water after each step and dried using ISO-size #40 paper points (Shanghai Dochem Industries, Shanghai, China) when the preparation was complete.

Post cementation was performed according to the manufacturer’s instructions. The posts were cleaned with a 96% alcohol solution before try in. If it fitted well, the post was cleaned once again with alcohol and dried. Afterward, the silanization of the post (Ceramic Bond, VOCO GmbH, Cuxhaven, Germany) was performed: the silanization of the post was performed for 60 s, and then the post was dried with oil-free air. Before cementation, the root canals were washed again with distilled water and then dried with ISO-size #40 paper points (Shanghai Dochem Industries, Shanghai, China). A bonding agent (Futurabond DC SingleDose, VOCO GmbH, Cuxhaven, Germany) was applied to the walls of the root canal for 20 s, and dried with same-size paper points. For the adhesive cementation, QuickMix Rebilda DC (VOCO GmbH, Cuxhaven, Germany) core build-up and luting cement were used. With the help of the intraoral tip, the luting material was applied directly into the root canals. After the insertion of the post, the cement was light-cured for 40 s with a visible light curing unit (O-Light, Woodpecker Medical Instrument Co., Guilin, Guangxi, China). All the specimens were stored in a distilled water container at 37 °C for one week.

### 2.3. Slicing of the Specimens for the Push-Out Test

**Figure 1:**
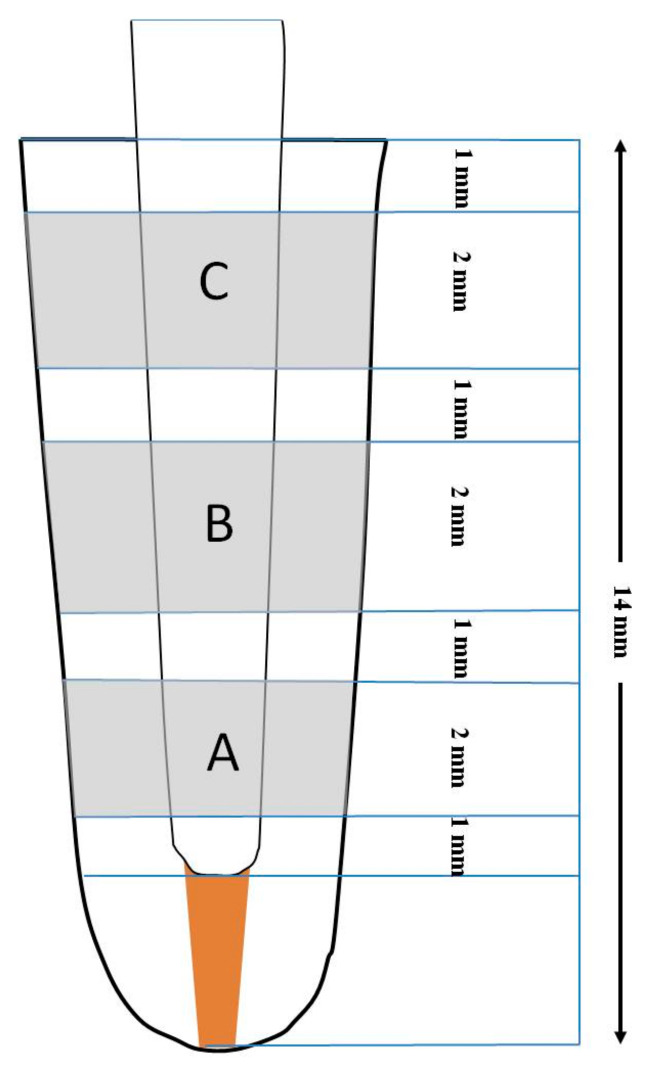
The cutting sequence followed to obtain the slices. A: apical section, B: middle section, and C: coronal section.

To evaluate the adhesion of the posts, each root was sliced into three sections with a 0.5 mm thick diamond-coated slow-speed band saw (Hager and Meisinger GmbH, Neuss, Germany) at a speed of 150 rpm under water-cooling from the apical, middle, and cervical regions of the root. The sectioning started 1 mm below the CEJ: there was a cervical (C), middle (B), and an apical (A) section with a thickness of 2 mm each. One slice from each region was selected at a depth of 1, 4, and 7 mm (Figure 1). Each section was marked on its apical surface.

Following the sectioning, 116 sample pieces were available. Four samples were fractured in the procedure, resulting in no measurements being taken with them. Each section was placed into a separated and labelled Eppendorf sample tube containing sterile saline, and delivered to Budapest University of Technology and Economics, Faculty of Mechanical Engineering, Department of Materials Science and Engineering, where the push-out tests were performed.

**Figure 2:**
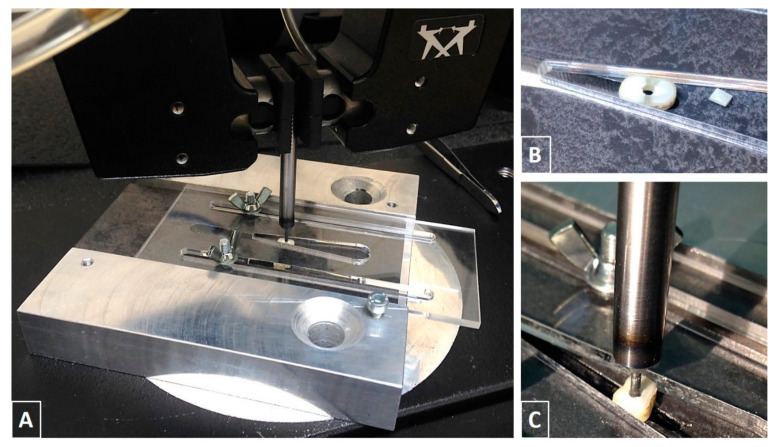
(**A**) A special holder designed for the examination. (**B**) A pushed-out post with a root section. (**C**) Steel rods used during the push-out tests.

The sections were individually fixed in a special holder, which was designed and produced specifically for this examination (Figure 2A). Thanks to the geometry and function of the holder, each geometry type of section was fixed in an optimal position and held there during the complete push-out test (Figure 2B). To push out the glass fiber-reinforced post, a steel (X210Cr12) rod with pin ends (Figure 2C) were used with various diameters, according to the fiber-reinforced post diameter (Table 2). The load was applied from the apico-coronal direction, avoiding limitations of post movement due to root canal tapering.

The push-out tests were performed with a commercial tensile test machine Instron^®^ 5965 (Instron, High Wycombe, Buckinghamshire, UK) with the following parameters: 2.5 mm/min crosshead speed, 5 kN Instron^®^ load cell, fixed-sample position, and manual start and end position. The forces as a function of the displacement were recorded in real-time, and Bluehill^®^ Testing software (v4.01) determined the peak force. The push-out tests were terminated when the glass fiber-reinforced posts fell out from the section. To calculate the bond strength in MPa, the recorded peak force (N) were divided by the area of the bonded surface, which was calculated using the following formula for each post [17]:

$$A=\pi \left(r_{1}+r_{2}\right)\sqrt{{\left(r_{1}+r_{2}\right)}^{2}+h^{2}}$$

where π was the constant 3.14, r_1_ was the coronal post radius, r_2_ was the apical post radius, and h was the thickness of the slice in millimeters. Table 3 shows the calculated surface (mm^2^) of the bonded area for each post segment.

### 2.4. Statistical Analysis

For the statistical evaluation of the data, SPSS analysis software (version 23.0; SPSS, Chicago, IL, USA) was used. The Kolmogorov-Smirnov test and Shapiro-Wilk test were used to check the distribution of the data. The Tukey post-hoc test and factorial ANOVA were used to compare the independent groups between the three regions of the root canal and between the four different post sizes.

## 3. Results

**Figure 3:**
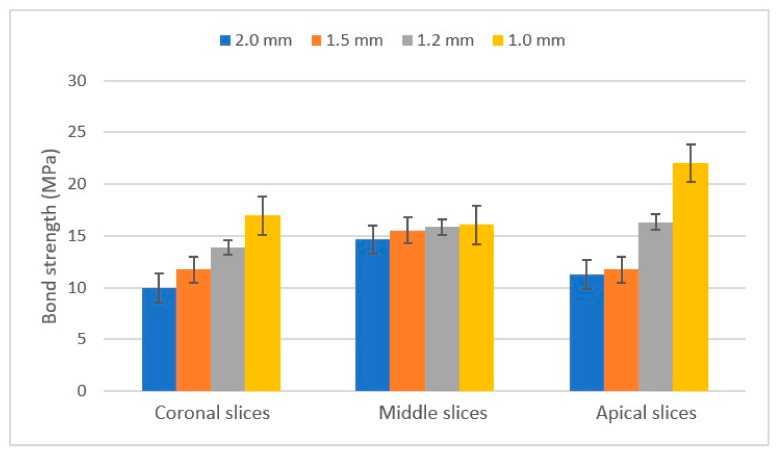
The mean push-out bond strength values (MPa) ± standard error of the mean (SEM) for each post.

A total sample size of n = 40 teeth was included in the study. Fiber-reinforced endodontic posts with four different diameters (10 each) were luted with a dual-cure resin-luting cement. Following sectioning, 116 samples were gathered and investigated through the push-out tests. The average recorded peak force (N) and mean push-out bond strength values (MPa) of the post groups in different root canal regions are shown in Table 4 and Table 5 and Figure 3.

The lowest peak force (N) values were obtained in the apical (A) post segments, while the highest values were in the middle (B) segments (excluding the 1.0 mm post). On average, the 2.0 mm posts had the highest peak value force with 111.99 ± 10.40 N, while the 1.0 mm posts had the lowest with 99.98 ± 8.05 N. The results were varied when the peak forces were divided by the surface of the bonded area of each post region. The average MPa value was the highest for the 1.0 mm posts (18.20 ± 1.67 MPa) and the lowest for the 2.0 mm posts (12.08 ± 1.05 MPa).

Both the Kolmogorov-Smirnov test and the Shapiro-Wilk test showed a normal distribution of data (*p* = 0.200 and *p* = 0.140, respectively). 

Comparing the sections of the posts showed no significant difference between the mean push-out bond strength values for the three groups (*p* = 0.219 for the factorial ANOVA analysis). Comparing the size of the posts, the factorial ANOVA showed a significant difference between the mean push-out bond strength values for the four groups (*p* = 0.002). The Tukey post-hoc test multiple comparisons test with a 5% significance level showed significant differences between the 1.0 mm–1.5 mm (*p* = 0.023) and 1.0–2.0 mm (*p* = 0.003) posts.

## 4. Discussion

In this in vitro study, the push-out bond strength (PBS) of fiber-reinforced posts was evaluated depending on the post diameter in the coronal, middle, and apical parts of the root canal. While many aspects of the post remained the same (form, adhesive and cementation protocol, and operative procedures), four different diameter posts (ø 1.0 mm, ø 1.2 mm, ø 1.5 mm, and ø 2.00 mm) were used. Push-out tests were used to measure the bond strength at the post-cementation junction. The results of these tests can be compared with the clinical conditions. The results of push-out tests are more reliable than conventional and modified microtensile tests in terms of the measurement of bond strength [17,18]. Thus, we used push-out tests to compare the bond strength of fiber posts with different diameters luted with the same preparation protocol and adhesive resin cement system to close out other potential influencing factors.

Our first null hypothesis stated that when the posts have different diameters, the retention of the posts will be the same. Comparing the diameters showed that the ø 2.00 mm posts had the highest mean push-out bond strength (111.99 ± 10.40 N), followed by the ø 1.5 mm (103.80 ± 10.32 N), ø 1.2 mm (101.00 ± 5.39 N), and ø 1.00 mm (99.98 ± 8.05 N) posts. The results were different when the peak forces were divided by the surface of the bonded area. According to our findings, the ø 1.00 mm posts had the highest mean push-out bond strength (18.20 ± 1.67 MPa), followed by the ø 1.2 mm (15.31 ± 0.85 MPa), ø 1.5 mm (12.86 ± 1.23 MPa), and ø 2.00 mm (12.08 ± 1.05 MPa) posts. The factorial ANOVA showed significant differences between the mean push-out bond strength values for the four groups (the significance level was *p* = 0.002). The Tukey post-hoc test with a 5% significance level showed a significant difference between the 1.0 mm–1.5 mm (*p* = 0.023) and 1.0–2.0 mm (*p* = 0.003) posts. Our study supports the findings of other authors: the bond strength of each post varies according to their diameter [19,20]. A study by Freitas and al. showed similar results: a group with higher adaptation to the root canal (thicker diameter posts) presented the highest bond strength results [21]. The higher bond strength results can be explained by the lower amount of resin cement needed to fill the space between the post surface and the dentin wall [21,22]. With less resin cement used, the polymerization shrinkage of the material is minimized and generates less stress on the bonding surface [21]. Resin cement has different physico-chemical properties (such as viscosity and flow), which are related to bond strength: materials with greater flow and lower viscosity can fill the spaces more perfectly, resulting in improved bonding [23,24,25,26]. Other factors, such as the post space, post surface treatment [27,28], and different post systems [29,30], contribute to the bond strength. From a practical point of view, a post with the most accurate fit should be luted inside the root canal [21] without overpreparation, weakening the reaming tooth and root structure [31], and optimizing the thickness of the luting cement [21,22].

The second null hypothesis stated that, along the length of the root canal, the bonding force between the canal and the post is equally distributed. When comparing the results, the middle segments had the highest push-out strength (116.21 ± 7.44 N), while the apical part (86.62 ± 7.42 N) had the lowest strength. The results were different when the peak forces were divided by the surface of the bonded area of each post region: the apical sections had the highest strength (15.62 ± 1.64 MPa), while the coronal part had the lowest strength (13.3 ± 0.86 MPa). The middle section push-out strength was 15.52 ± 0.92 MPa. Comparing the regions of the posts showed no significant difference between the mean push-out bond strength values for the three groups. The *p*-value was 0.219 for the factorial ANOVA analysis. Our results are similar to previous studies: no statistical difference was found among the root canal regions [22,26], while the lowest bond strength was achieved in the apical region, with the highest in the middle region [32]. The lower bond strength in the apical segments can be explained by the difficulty of the proper bonding procedure (applying and drying the proper amount of bonding agent), as well as the increased dentin wall–post surface space due to the evasion of the drill, which would lead to a thicker cement layer and increased polymerization shrinkage [32].

Other factors contributing to the retention of posts are well studied [1,7,14,15]. However, a limited number of pieces of literature were found where the study focused on different diameters of the same post system [19,20,21] and root regions [22,26,31]. The effects of different diameters of posts and root regions still need to be researched more to make an adequately reliable conclusion.

## 5. Conclusions

The present in vitro study was conducted to evaluate the push-out bond strength (PBS) of a single system of glass fiber-reinforced composite posts (Rebila, VOCO, Cuxhaven, Germany) with four different diameters (1.0, 1.2, 1.5, and 2.0 mm) in three regions of the root canal (apical, middle, and coronal). 

In our study, there was a significant statistical difference among the bond strength of the different diameters of the posts (*p* = 0.023 between the 1.0–1.5 mm posts and *p* = 0.003 between the 1.0–2.0 mm posts). The 2.0 mm posts had the highest PBS (average 111.99 ± 10.40 N), which was followed by the 1.5 mm, 1.2 mm, and 1.0 mm posts. The bond strength differed when the bonded area of the posts was calculated: the 1.0 mm posts had the highest MPa value (average = 18.20 ± 1.67 MPa). A further increase in the diameter showed a decrease in the PBS calculated on the bonding surface. The study found no statistical difference between the root canal regions in bond strength. 

A limitation of our study was that the measurement of PBS was performed in a model system, not in vivo. More samples with different diameters could have supplied more precise values. Because of this limitation of our study and the lack of other studies related to this topic, more research and data are needed on this topic: further investigation should be carried out on other post types with different diameters with increased sample sizes to determine an optimal choice of size during post and core treatments. 

## Figures and Tables

**Table 1:** Instrumentation of each group according to the manufacturer (drill marked with “+” was used, while with “-“ wasn’t used in a given group).

Group Number	1 (1.0 mm)	2 (1.2 mm)	3 (1.5 mm)	4 (2.0 mm)
Ø 0.7 mm reamer	+	+	+	+
Ø 1.0 mm drill	+	+	+	+
Ø 1.2 mm drill	-	+	+	+
Ø 1.5 mm drill	-	-	+	+
Ø 2.0 mm drill	-	-	-	+

**Table 2:** Pin diameters according to the post diameters.

Pin diameters (mm)	0.5	0.7		1.0
Fibre-reinforced post diameters (mm)	1.0	1.2	1.5	2.0
Root sections	A	B		C

**Table 3:** Surfaces of the bonded area (mm^2^) for each post.

Section/Diameter	1.0 mm	1.2 mm	1.5 mm	2.0 mm
“A” section	4.43	5.25	6.32	7.58
“B” section	5.94	6.98	8.08	9.34
“C” section	6.28	7.54	9.32	11.06

**Table 4:** The average recorded peak force (N) for each post (including the standard error of the mean (SEM)).

	Peak Force (N)			
Section/Diameter	1.0 mm	1.2 mm	1.5 mm	2.0 mm
“A” section	97.58 ± 19.45	85.70 ± 12.18	74.16 ± 16.59	85.46 ± 10.45
“B” section	95.42 ± 14.54	110.66 ± 6.55	125.40 ± 20.69	136.82 ± 16.13
“C” section	106.64 ± 8.92	104.72 ± 7.96	109.53 ± 13.77	110.13 ± 22.30
Average	99.98 ± 8.05	101.00 ± 5.39	103.80 ± 10.32	111.99 ± 10.40

**Table 5:** The mean push-out bond strength values (MPa) for each post (including the standard error of the mean (SEM)).

	Mean Push-Out Bond Strength (MPa)			
Section/Diameter	1.0 mm	1.2 mm	1.5 mm	2.0 mm
“A” section	22.03 ± 4.39	16.32 ± 2.32	11.73 ± 2.63	11.27 ± 1.38
“B” section	16.06 ± 2.45	15.85 ± 0.94	15.52 ± 2.56	14.65 ± 1.73
“C” section	16.98 ± 1.42	13.89 ± 1.06	11.75 ± 1.48	9.96 ± 2.02
Average	18.20 ± 1.67	15.31 ± 0.85	12.86 ± 1.23	12.08 ± 1.05

## Data Availability

The data are available upon request from the corresponding author.
